# Maternal exposure to zinc oxide nanoparticles causes cochlear dysfunction in the offspring

**DOI:** 10.3389/ftox.2024.1323681

**Published:** 2024-01-11

**Authors:** Luisa Campagnolo, Valentina Lacconi, Roberta Bernardini, Andrea Viziano, Antonio Pietroiusti, Lorenzo Ippoliti, Arturo Moleti, Renata Sisto

**Affiliations:** ^1^ Department of Biomedicine and Prevention, University of Rome Tor Vergata, Rome, Italy; ^2^ Department of Clinical Sciences and Translational Medicine, University of Rome Tor Vergata, Rome, Italy; ^3^ Department of Physics, University of Rome Tor Vergata, Rome, Italy; ^4^ Unicamillus University of Medical Sciences, Rome, Italy; ^5^ Department of Occupational and Environmental Medicine, Epidemiology and Hygiene, Italian Workers Compensation Authority, Rome, Italy

**Keywords:** ZnO nanoparticles exposure, fetotoxicity, cochlear function, offspring, distortion product otoacoustic emissions

## Abstract

**Introduction:** Zinc oxide nanoparticles (ZnO NPs) have been engineered and are largely used in material science and industry. This large and increasing use justifies a careful study about the toxicity of this material for human subjects. The concerns regard also the reproductive toxicity and the fetotoxicity.

**Materials and methods:** The effect of the exposure to ZnO NPs on the cochlear function was studied in a group of pregnant CD1 mice and in their offspring. This study is part of a larger toxicological study about the toxicity of ZnO NPs during pregnancy. Four groups were analyzed and compared, exposed and non-exposed dams and their offspring. The cochlear function was quantitatively assessed by means of Distortion Product Otoacoustic Emissions (DPOAEs).

**Results and discussion:** A large statistically significant difference was found between the non-exposed dams offspring and the exposed dams offspring (*p* = 1.6 · 10^−3^), whose DPOAE levels were significantly lower than those of non-exposed dams offspring and comparable to those of the adults. The DPOAE levels of the exposed and non-exposed dams were very low and not significantly different. This occurrence is related to the fact that these mice encounter a rapid aging process.

**Conclusion:** Our findings show that maternal exposure to ZnO NPs does not reflect in overt toxicity on fetal development nor impair offspring birth, however it may damage the nervous tissue of the inner ear in the offspring. Other studies should confirm this result and identify the mechanisms through which ZnO NPs may affect ear development.

## 1 Introduction

The physico-chemical properties of nanoparticles justify their wide use in diverse materials and products. Zinc oxide nanoparticles (ZnO NPs) have been engineered and are largely used in material science and industry. Zinc oxide nanoparticles (ZnO NPs) are among the most widely used nanoparticles due to their unique properties that make them components for pigments, semiconductors and sensors, potential theranostic agents and additives for cosmetics and food (Raha and Ahmaruzzaman, 2022). Although ZnO NPs are widely used, some concerns have arisen about the risk for humans coming from exposure through different routes: skin absorption, ingestion, inhalation, etc. (Pietroiusti et al., 2018a).

Recent studies (Hou et al., 2018) showed how the ZnO NPs can exert a toxic effect on a large variety of biological systems, plants, invertebrates, vertebrates and microorganisms. *In vitro* and *in vivo* studies were performed in order to clarify the toxicity mechanisms. The findings of these studies suggest that toxic effects are due to inflammation, apoptosis and oxidative stress (Campagnolo et al., 2012). The neurotoxic effects of ZnO NPs seem to be well assessed. Indeed, *in vitro* studies have shown that ZnO NPs may be toxic to astrocytes and neuronal cells, by inducing oxidative stress and mitochondrial dysfunction, and by perturbing the expression of proteins and miRNA involved in neuronal differentiation Sudhakaran, 2019; Srivastava et al., 2020). The neurotoxic effect of ZnO exposure has been also investigated *in vivo* in rodents. Repeated intraperitoneal administration of the particles in rats resulted in the attenuation of spatial learning and memory ability (Han et al., 2011), while repeated intranasal instillation showed alterations in sniffing behavior as a consequence of damage to the olfactory epithelium, as well as oxidative damage, inflammatory responses, and histopathological alterations in the olfactory bulb, hippocampus, striatum, and cerebral cortex (Gao et al., 2013; Liu et al., 2019). These results support the hypothesis that ZnO NPs administered through different routes and at different doses may damage the nervous tissue. Due to their small dimensions NPs have the capability of crossing the placental barrier causing toxic effects to the fetus during intrauterine development. A complete review about fetotoxicity induced by NPs can be found in Teng et al. (2021).

Several experiments were performed in order to assess if the maternal exposure could induce a risk for pregnancy or fetal development.

A large bibliometric analysis on the topic of the reproductive and fetal developmental toxicity of nanoparticles is reported in Wang et al., 2018. The engineered nanomaterials are supposed to have different toxicity mechanisms as regards the effects on fetal development and the effect on the reproductive capability. These different mechanisms are related to the physico-chemicals characteristics of the nanomaterials (Campagnolo et al., 2012). Several studies have investigated the reprotoxic effect of ZnO NPs of different size, administered to pregnant rodents. Chen et al. observed *in utero* growth restriction and reduced number of fetuses following maternal oral exposure to 30 nm ZnO NPs at relatively high doses (the lowest dose being “hundred times as much of dietary reference intakes for zinc recommend by the Chinese nutrition society”) and at later stages of pregnancy (GD10.5-GD17.5) (Chen et al., 2019). In another study, Teng et al. reported a size-dependent translocation of the placental barrier, as smaller particles of 13 nm were reaching the fetal tissue and induce toxicity, while bigger particles of 57 nm were not. This study also identified later stages of gestation (GD7-16) being more sensitive than earlier stages (GD1-10) for what concerns fetal viability and growth. These results are in line with the results of the present study in which no differences were observed in the number of fetuses per females or of pups per litter between dams that were exposed or non-exposed to ZnO NPs soon after embryo implantation. Overall, these observations highlight that the toxicity of ZnO NP may depend on different parameters and careful should be paid in drawing conclusions. Different studies, with different types of NPs and different outcomes are discussed in Hougaard, 2015. Interestingly, toxicity may derive from the NPs reaching the placenta and translocating the placental barrier; however the adverse effect on fetal development may also derive indirectly from the particles affecting placental functions, with no placental barrier translocation. In the present study, translocation of particles across the placenta and fetal deposition were not assessed, however from our previous studies (Pietroiusti et al., 2018b) and studies form other groups it has been shown that nanoparticles may affect fetal development also by placental mediated indirect effects (Hougaard, 2015; Dugershaw, 2020). The exposure during the prenatal period to ZnO NPs was found associated to intrauterine fetus growth retardation (Teng et al., 2020), decreased fetal weight, altered blood pressure in offspring (Morales–Rubio et al., 2019). Neuro fetotoxicity has been also found associated to the exposure to ZnO NPs during the gestational period. In particular, the development of neurological defects, such as learning and memory impairment (Feng et al., 2017), motor coordination deficits and depressive-like behavior (Alimohammadi et al., 2019a; Alimohammadi et al., 2019b) has been reported.

The present *in vivo* study was aimed at investigating the possible toxic effect of intravenous injected ZnO NPs on pregnant dams and fetal development after maternal exposure during pregnancy. In order to investigate the neurological effect of the exposure both on the pregnant dams and their offspring, the auditory receptor was chosen as target organ. The cochlea is, in fact, very sensitive to the exposure to different xenobiotics, and the hearing function alteration was already used in our previous studies as a very early biomarker of toxicity both in animals (e.g., Fetoni et al., 2022, using the same instrument of the present study) and in humans (Jusko et al., 2014; Sisto et al., 2022).

The alterations in cochlear function can be diagnosed by means of Otoacoustic Emissions (OAEs), acoustic signals that can be measured in the ear canal as a by-product of the active amplification mechanism occurring in the cochlea and mediated by the Outer Hair Cells (OHC). As a by-product of the development of an amplified forward traveling wave that focuses the stimulus energy of each frequency component within a specific tonotopic region causing frequency specific perception, a backward traveling wave is generated in the cochlea by a set of different mechanisms. The associated pressure signal (the OAE) is measurable in the ear canal using a low-noise acoustic measurement chain. OAEs can be elicited by different stimuli. In this study, Distortion Product OAEs (DPOAEs) were used. DPOAEs are the nonlinear response elicited by two tones of nearby frequencies *f*_1_ and *f*_2_ at intermodulation frequencies. The most intense DPOAE, measured in the present study, occurs at frequency *f*_dp_ = 2*f*_1_–*f*_2_.

As regards the relation between neurotoxicity and DPOAEs, a study performed on patients affected by Parkinson disease (Garasto et al., 2023), shows that the DPOAEs are significantly associated to dopamine transporter availability. This association was showed by measuring the dopamine transporter levels in the brain nuclei contralateral with respect to the ear from which the DPOAEs were recorded, demonstrating the association between the dopaminergic neurons and DPOAEs in humans.

In the present study, we show that maternal exposure to well characterized and standardized ZnO NPs does not reflect in overt toxicity to the developing fetuses; however, after birth, the progeny presents alteration in the DPOAE measurements, demonstrating insidious potential toxicity of these nanoparticles.

## 2 Materials and methods

### 2.1 Nanoparticle characterization and suspension

ZnO nanoparticles NM111 were obtained from the JRC repository. Detailed physical-chemical characterization of the particles used in this study is provided in the JRC reports (Singh et al., 2011). These particles have been also used in a previously published study, where characterization of the suspensions is reported (Farcal et al., 2015). According to Singh et al. (2011) the ZnO NM-111 suspension, quantitatively analyzed by means of transmission electron microscopy (TEM), has a mean primary particle diameter (Feret’s mean diameter) of approximately 141 nm (median = 119 nm, 25% percentile = 68 nm, 75% percentile = 189 nm).

The ZnO NM -111 consists of both spherical and non-spherical primary particles, with wide size-range. NM-111 is coated with triethoxycapryl silane and is hydrophobic. The mean aspect ratio is 1.8 (median = 1.6, 25% percentile = 1.4, 75% percentile = 2). The statistics relative to 26 parameters related to the particles morphology is shown in Singh et al. A stock suspension ZnO NPs was prepared at a concentration of 1 mg/mL in 0.05% BSA in deionized water. The NP suspension was then sonicated on ice for 3 min at 800 W (40% amplitude) using a 3 mm probe (Branson Digital Sonifier, Danbury, Connecticut, United States), and kept on ice and vortexed for 1 min right before use. The working suspension was prepared by diluting the stock suspension in saline solution. ZnO NPs were handled following standard operating procedures described in the JCR reports. The particle suspension was prepared under sterile conditions and the presence of any potential bacterial contamination was assessed in parallel by incubating part of the suspension at 37°C and monitoring the presence of bacterial growth over time under the microscope. Moreover, the presence of endotoxins was tested by the use of an endotoxin quantitation kit (Pierce Chromogenic Endotoxin Quant Kit, Catalog n. A39552).

### 2.2 Animal exposure

**FIGURE 1:**
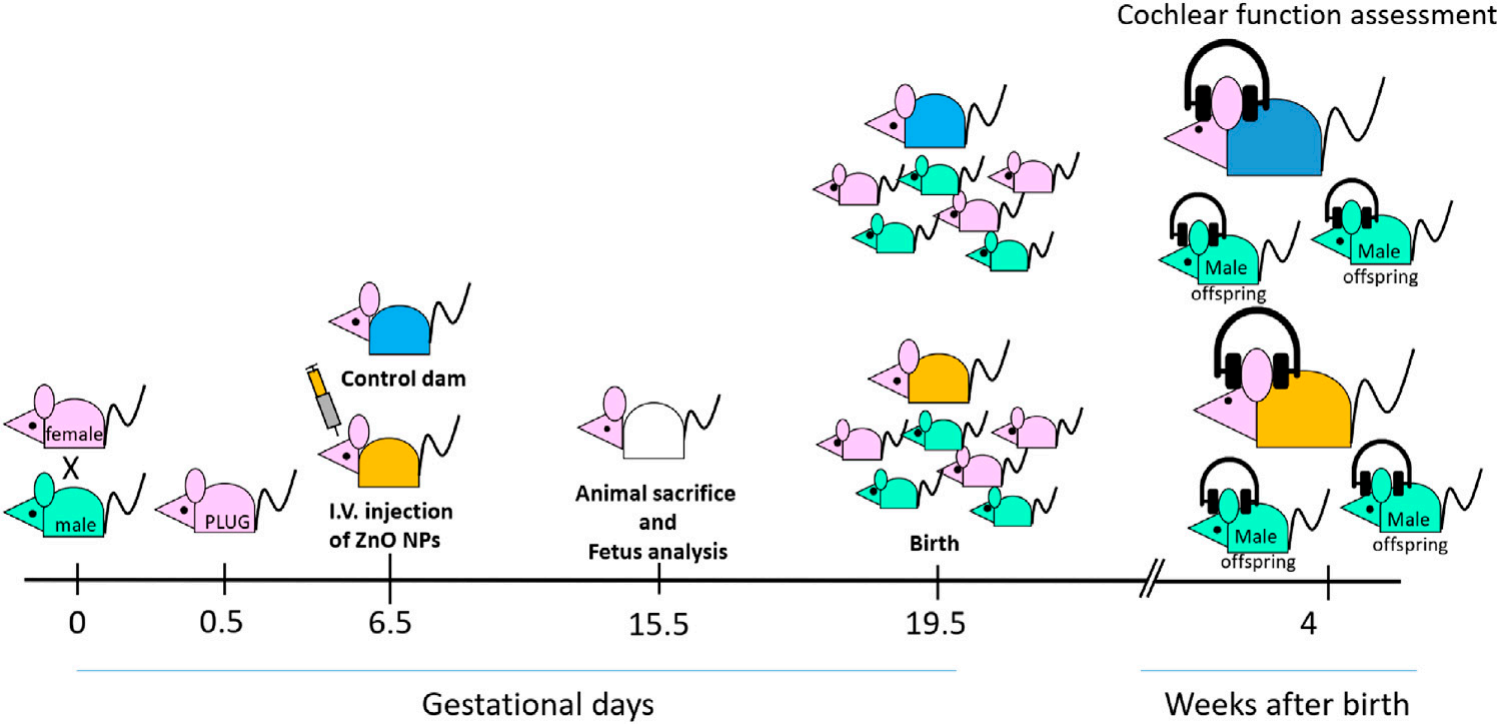
Timeline of the experiment.

Eight-week-old CD1 female mice were obtained from Charles River Italia (Calco, LC, Italy) and housed in the Tor Vergata Animal Technology Station under standard conditions (25°C, 50% relative humidity) on a 12 h light/dark cycle with free access to water and food. All animal procedures were in compliance with the European Legislation (2010/63/EU) and were approved by the Institutional Animal Care and Use Committee of the University of Tor Vergata and by the Italian Ministry of Health (Auth. no 262/2021-PR). A veterinary surgeon, responsible for the welfare of laboratory animals, was present during all sets of experiments. Animal care was under the responsibility of trained personnel. Mice were divided in groups and the number of mice in each group was calculated by the G Power Analysis. The objective was that of using as few animals as possible while still obtaining meaningful results. The G*Power 3.1 power analysis software was used to calculate the size of each treatment group considering to see a mean difference of 50% between the groups, 80% probability of detecting this difference and *α* error to 0.05. Thirty pregnant mouse females (GD6.5) were divided into 3 groups, which received 0, 0.1 or 1 mg/kg of ZnO NPs. The dosage administered to pregnant mice in this study has been selected on the basis of our previously published data using other types of metal nanoparticles which showed no evident sign of toxic effects on the pregnant females and on the offspring up to the dose of 1 mg/kg of body weight (Pietroiusti et al., 2018a). Since we were interested in evaluating the potential subtle effects of ZnO nanoparticles on development which may manifest after birth, we chose a dose that had no major toxic effects on the developing fetus and that would allow the birth of the offspring and the DPOAE measurements. Half of the females were sacrificed at GD15.5 and the number of fetuses/female and their size and morphology were evaluated under a dissection microscope. The other half of females was allowed to give birth. For the cochlear function testing, only females receiving the highest dose of ZnO NPs were included. The DPOAEs measurement was performed when the mothers were 12 weeks old, and the offspring was 4 weeks old. Specifically, on GD 6.5 each mouse received 100 μL of a suspension containing 1 mg/kg of body weight of ZnO NPs by intravenous injection in the retro-orbital plexus, as previously reported (Pietroiusti et al., 2018a). Although intravenous injection is not a primary route of nanoparticle exposure, in the present study it was decided to deliver the ZnO NPs in order to directly correlate the administered dose to possible toxic effects. Indeed, by being directly delivered to the blood stream, NPs do not have to cross the primary intestinal or lung barriers. Moreover, this route of administration would give indication on the effects following potential biomedical applications of ZnO NPs. Control females were injected with 100 μL saline solution (vehicle). For the DPOAE measurements, 5 pregnant females non-exposed and 5 pregnant females exposed to ZnO NPs were allowed to give birth. Eight 4-week-old male mice, randomly selected among the offspring of ZnO NP exposed mothers and seven from non-exposed mothers were then used for the DPOAE measurements. Both the right and left ear of each individual were tested. DPOAE measurements were performed under general anesthesia by intra-abdominal injection of tiletamine/zolazepam (30 mg/kg bw Zoletil 100, Virbac, Italy) associated to xylazine (5 mg/kg bw Rompun, Bayer, Italy). A timeline plot of the experiment is shown in Figure 1.

### 2.3 DPOAE measurement

DPOAEs were measured using a custom acquisition system based on 24-bit NI-4461 PXI data acquisition boards, programmed in Labview (National Instruments, United States), driving two ER-2 loudspeakers and recording the response of an ER10B + low-noise microphone (Etymotic Research, United States). High-resolution 2*f*_1_-*f*_2_ DPOAE complex spectra were recorded using chirp stimuli, with a constant primary frequency ratio *f*_2_/*f*_1_ = 1.22, stimulus levels (L_1_, L_2_) = (61, 55) dB SPL, in the range *f*_DP_ = 4–12 kHz, with 20Hz frequency resolution. Two mechanisms are involved in the DPOAE generation: the nonlinear distortion occurring close to the f2 tonotopic place, the linear reflection occurring at the *f*_dp_ tonotopic place, characterized by lower amplitude and longer latency. A time-frequency wavelet filtering method was applied (Moleti et al., 2012), to unmix these two DPOAE components according to their different phase-gradient delay, which is the theoretically predicted signature of the different OAE generation mechanisms, nonlinear distortion and linear coherent reflection (Shera and Guinan, 1999; Kalluri and Shera, 2001). This filtering technique yields two spectra, one containing the distortion, or zero-latency (ZL) component, the other containing the reflection, or long-latency (LL) component. An advantage of the unmixing process is to increase significantly the signal-to-noise ratio (by 5–15 dB, particularly in the high-frequency range). After separating the DPOAE components, the signal level was computed in third-octave frequency bands. A signal rejection criterion was applied to exclude from the analysis, for each spectral band, the recordings with average noise level exceeding significantly the instrumental noise floor.

### 2.4 Statistical analysis

Statistical analyses of the parameters of the fetuses and of the offspring were performed with Statistics Kingdom (2017) software. Ordinal variables are presented as mean ± SD (Standard Deviation). The normal distribution of data was checked with the Shapiro-Wilk test: all data are normally distributed. For comparison between two groups, we performed the *t*-test for normal data.

DPOAE statistical analysis was performed by means of the R version 4.0.3 (2020-10-10), Copyright (C) 2020, The R Foundation for Statistical Computing, Vienna, Austria. A four level factor “group” was introduced, distinguishing the different categories of exposed and unexposed mothers and their offspring. ME and MC stands, respectively, for the mothers exposed and unexposed during pregnancy, SC indicates the offspring of unexposed mothers and SE refers to the offspring of the mothers exposed during pregnancy.

As repeated measures of the DPOAEs at different frequencies (five 1/3 octave bands) were performed in the same subject, the repeated measure ANOVA test was performed with the aim of assessing the significance of the factor “group”. The multiplicity due to the measurements taken on the two ears of the same subject was also taken into account. A *post hoc* analysis was also performed at the aim of studying what are the categories that differ at a statistically significant level inside the factor group.

Mixed effect linear regression models (nlme) were also studied with the objective of quantitatively determining the effect of group on the DPOAE amplitudes at different frequencies. At this aim the DPOAE amplitudes were arranged in a unique vector and a factor variable, named “freq”, was created labeling the different frequency bands.

The models were of the kind:

$$\text{lme}\left(\text{ DPOAE }∼\text{ group}*\text{freq}+\text{random}\left(\;∼1|\text{subj}\right)\right),$$

where freq and group are the fixed effect factors of interest. The subjects were treated as random variable and random intercepts were fitted. The model fitting was stopped when the minimum AIC and BIC indexes were obtained. As the mixed-effect models do not produce a determination coefficient, the statistical significance of the model itself was tested by fitting two models, one including the fixed effect factor of interest and the other one without it. The two models were compared by means of an ANOVA test. A standard significance criterion *p* < 0.05 was assumed.

## 3 Results

### 3.1 Effects of ZnO NPs on fetal development

Five pregnant mouse females in each group were sacrificed at GD 15.5 and fetal parameters recorded. The exposure to 0, 0.1 and 1 mg/kg ZnO NPs early in gestation (GD 6.5) did not result in statistically significant differences in the number of fetuses and fetal resorptions per female, and in fetal crown-rump length. Results are summarized in Table 1.

**TABLE 1:** Effects of maternal exposure to ZnO NP on fetal development.

	CTRL	NM-111 0.1 mg/Kg bw	NM-111 1 mg/Kg bw
Pregnant females	5	5	5
Fetuses/female	14.2 ± 1.48	14.4 ± 1.52	14 ± 2.91
Total resorptions	1	1	0
Crown-rump length (mm)	15.22 ± 0.72	15.24 ± 0.39	15.08 ± 0.92

No statistically significant differences were detected among groups.

### 3.2 Effects of ZnO NPs on the offspring

Five pregnant females were allowed to give birth and no statistically significant differences in the number of pups per litter, nor in the sex ratio or the survival rate between groups were observed (not shown). At the age of 4 weeks, the male offspring of dams exposed to the highest dose of 1 mg/kg of ZnO NPs (SE) and those (SC) from control dams were weighted and randomly selected for DPOAE measurements. No differences were observed in terms of weight among the two groups (27.92 ± 0.30 and 28.1 ± 0.35 g, SC and SE, respectively; *t*-test *p* = 0.17). No statistically significant differences were also observed in the weight of the ZnO NP exposed and non-exposed dams (30.6 ± 1.2 g and 31 ± 1.5 g, ME and MC, respectively; *t*-test *p* = 0.6).

When the offspring was subjected to DPOAEs measurement, the factor group in the repeated measure ANOVA was found statistically significant (*p* < 10^–4^). A *post hoc* analysis with Bonferroni-Holm correction showed that the DPOAEs of the unexposed dams offspring are significantly larger than the DPOAEs of the exposed female offspring (*p* = 1.6 · 10^−3^). The DPOAE levels of the SC group were also significantly larger than those of the pregnant females, both exposed (*p* = 1.6 · 10^−3^) and unexposed (*p* = 8·10^−4^), i.e., groups ME and MC respectively. All other group comparisons, and, in particular that between the exposed and unexposed dams, ME and MC, were not significant. No effects related to the ear side were found.

**FIGURE 2:**
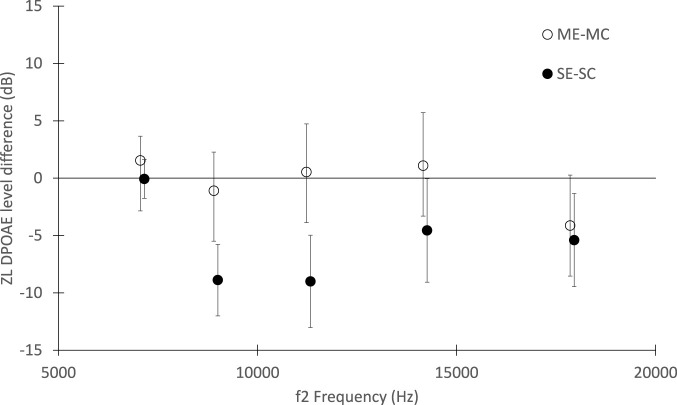
Distortion Product Otoacoustic Emissions level differences between exposed and unexposed groups, as function of the *f*_2_ stimulus frequency. The differences between the exposed and control mothers and between the offspring of the exposed and unexposed mothers are shown (white and black circles respectively). The standard errors are also shown.

In Figure 2, the average DPOAE level differences between exposed and unexposed groups are shown in five 1/3 octave bands. The significant effect of the dams’ exposure during the gestational period on the offspring hearing is visible, particularly in the mid-frequency range (*f*_2_ = 8–12 kHz), while no difference was observed between exposed and unexposed dams. No effects related to the ear side, left or right, were found.

## 4 Discussion

Although, under the exposure conditions chosen for this study, no significant effects on the number of pups per litter and in pups’ weight were observed, differences in the DPOAE measurements between exposed and non-exposed offspring appeared to be statistically significant, suggesting that exposure to ZnO NPs during development induce alteration in the auditory system that may manifest later in life, and indicate that DPOAE measurement may be a quantitative biomarker of toxicity.

The first finding is in agreement with other studies in literature. Zhai and coworkers (Zhai et al., 2018) did not observe any effect on the number and weight of the progeny after maternal intravenous administration of much higher doses (16 mg/kg of body weight) of ZnO NPs of size similar to the particles that were used in the present study. In another study, Chen et al. (2020) administered ZnO NPs via gavage at doses of 20, 60, 180 and 540 mg/kg bw and observed fetal toxicity with a dosage of 180 mg/kg bw and above; considering that it has been estimated that gut translocation of nanoparticles occurs between 0.01% and 3%, it can be assumed that the dose we administered to the pregnant mice is in the range expected to reach the blood stream in the above mentioned study. Another study in rats (Lee et al., 2016) following intravenous injection of ZnO NPs reports the NOAEL (No Observed Adverse Effect Level) to be 10 mg/kg for fetal developmental toxicity. According to the EFSA (European Food Safety Authority, 2015) the NOAEL for ZnO would be 50 mg/day, and an upper limit of 25 mg/person per day was recommended. The dose tested in the present study are comparable with the EFSA NOAEL, although it must be noted that these last are referred to the ionic state ZnO and not to the ZnO NPs.

This is the first study, to our knowledge, in which the neurotoxic effect on the fetal development of the ZnO engineered NPs was quantified by means of a biomarker of cochlear functionality, i.e., the Distortion Product Otoacoustic Emissions. The only other outcome found in literature is the acoustic startle which could be affected by deafness but also by central nervous system dysfunction.

The main result of this study is that the DPOAE levels of the offspring of unexposed dams are significantly higher than those of the offspring of exposed dams, while no significant difference was found between the DPOAE levels of exposed and unexposed dams. This last result could be due to the fact that the hearing function in these animals rapidly declines with adulthood (Riva et al., 2007) and, consequently, the DPOAE levels are not significantly different in exposed and unexposed adult mice. The proposed biomarker could be used in order to assess a dose-response curve in which the outcome variables are the DPOAEs amplitudes. In fact, whilst, for example, the acoustic startle is described by a dichotomous variable, the DPOAEs are a continuous variable, much more useful from the point of view of establishing a relation with the dose.

On the other hand, the present study suffers from several limitations. First of all, it was conducted on a small number of individuals and our evidences on the DPOAE levels should be confirmed on a larger data set. Another limitation comes from the fact that no extensive DPOAE normative data base had been collected with the instrument used in this study on CD1 mice of different age, so the conclusions of this study are based only on the differences observed between groups of the same age. In addition, CD1 mice develop presbycusis quite early (Riva et al., 2007), so. Although they are a standard choice for toxicologic studies, they could be a non-optimal choice for comparing the DPOAE levels in adult individuals. The toxicity mechanism is unknown, therefore we do not have a mechanistic model for predicting what DPOAE frequency bands should be most affected by the exposure.

## 5 Conclusion

A statistically significant difference was observed in the DP-gram of the offspring of unexposed (SC) and exposed dams (SE). This observation, if confirmed, would suggest the hypothesis that the exposure to ZnO NPs, at mild dose, of the order of magnitude of the EFSA NOAEL, could exert during the gestational period an effect on the fetus development and, in particular, on the development of the peripheral auditory system. The inner ear damage in the offspring of exposed dams could be considered as an evidence of the neurotoxicity of the ZnO oxide NPs in the fetal development. The use of a quantitative biomarker such as the DPOAE level is, in our opinion, a significant improvement in the research about the toxicity during the pregnancy and the effects of the exposure on the offspring. On the other hand, we remark again that the presented results should be interpreted with caution. First of all, the results are referred to a dose administration at the early stage of the gestational time. In addition, the fetotoxicity found in this study is strictly related to the ZnO NPs NM111 of the JCR repository, the toxicity effect being strongly related to the particle size distribution.

## Data Availability

The raw data supporting the conclusion of this article will be made available by the authors, without undue reservation.
